# Reducing inappropriate lumbar MRI referrals: a Danish pre- and post-intervention study

**DOI:** 10.1186/s12998-026-00663-x

**Published:** 2026-07-04

**Authors:** C. Bell, S. B. Krogh, P. Vedsted, R. K. Jensen, M. Fenger-Grøn, T. S. Jensen

**Affiliations:** 1 University Clinic for Innovative Patient Pathways, Medical Diagnostic Center, Regional Hospital Central Jutland, Silkeborg, Denmark; 2 Research Unit for General Practice, Aarhus University, Aarhus, Denmark; 3 Department of Sports Science and Clinical Biomechanics, University of Southern Denmark, Odense, Denmark; 4 Chiropractic Knowledge Hub, Odense, Denmark; 5 Diagnostic Imaging Department, Regional Hospital Central Jutland, Viborg, Denmark

**Keywords:** Referrals, Magnetic resonance imaging, Lumbar spine, Low back pain

## Abstract

**Background:**

Low back pain (LBP) is the leading cause of disability worldwide. Despite guidelines advising against routine imaging, a significant proportion of patients are referred for lumbar spine magnetic resonance imaging (MRI) without an appropriate indication. This study aims to assess the impact of the intervention on the appropriateness of referrals and the proportion of referred patients from general practitioners (GPs) who underwent lumbar MRI.

**Methods:**

A quasi-experimental design with pre- and post-intervention periods was employed. We included GP referrals for lumbar spine MRI at Silkeborg Regional Hospital, Denmark, among adults ≥ 18 years from 1 January 2019 to 31 August 2023. The intervention, which consisted of template-based correspondence letters and a guidance booklet, was implemented on 1 September 2022. Referrals were classified as either ‘appropriate’ or ‘inappropriate’ according to international guidelines. Interrupted time-series analysis with segmented regression was applied to evaluate change over time in referral appropriateness and the proportion of lumbar MRIs within 90 days of referral.

**Results:**

A total of 5222 referrals were received (80.6% pre and 19.4% post intervention). Patient characteristics were comparable across the periods. The proportion of appropriate referrals increased from 49.7 to 60.0%. At the onset of the intervention, the level of appropriate referrals increased by 6.50% points (95% CI 0.71, 12.29), and the trend changed by 0.98 (95% CI 0.36, 1.59), reaching 0.90 (95% CI 0.30, 1.49). This implied a continued increase in the pre-post difference.

The proportion of referred patients having lumbar MRIs decreased from 92.2 to 75.7%. At intervention onset, the level of lumbar MRIs changed by − 17.06 (95% CI − 23.25, − 10.88). However, post-intervention the trend changed, becoming positive (0.66, 95% CI − 0.05, 1.37), implying that the number of lumbar MRIs could return to pre-intervention level over time.

**Conclusion:**

The intervention increased the proportion of appropriate referrals for lumbar MRI. For the proportion of MRIs performed, we found an immediate reduction, but this may not be sustained over time.

**Supplementary Information:**

The online version contains supplementary material available at https://doi.org/10.1186/s12998-026-00663-x.

## Background

Low back pain (LBP) represents a major global health burden, with an estimated prevalence exceeding 500 million individuals worldwide, and it is the leading cause of disability worldwide [1]. Due to anticipated demographic shifts, the number of individuals affected by LBP is projected to increase by 36% by the year 2050 [1]. The aetiology of LBP is multifactorial, and in addition to identifiable pathological causes, several modifiable risk factors such as sedentary behaviour, smoking, obesity, and low socioeconomic status can contribute to the occurrence and progression of LBP [1, 2].

The vast majority of LBP patients (80–90%) have no indication of a serious pathological cause for their back pain and should be managed with conservative treatments, non-surgically, including guidance and reassurance, patient education, exercise, physiotherapy, chiropractic care, cognitive behavioural therapy, or pain management [3, 4]. The remaining 10–20% of LBP patients will not recover completely from initial management or may have signs and symptoms that raise suspicion of pathology. For this smaller group, international imaging guidelines recommend that they may be referred for diagnostic imaging, including magnetic resonance imaging (MRI) [4–8]. The same guidelines recommend against routine imaging for non-specific LBP, as MRI often cannot identify the cause of pain, and there is little evidence that it helps guide treatment decisions or contributes meaningfully to outlining the patient’s prognosis. Routine use of imaging can lead to unnecessary diagnostic workup and treatments without yielding a better outcome for the patient [9–11]. According to Danish national “Choosing Wisely” recommendations, MRI can be considered if other relevant treatments have been attempted for 4–6 weeks without effect or if there is suspicion of serious underlying pathology [12, 13].

There is compelling evidence that a significant proportion of patients with LBP are subjected to inappropriate healthcare practices that deviate from established guidelines [14–16]. The proportion of inappropriate referrals for lumbar spine MRI from Danish general practitioners (GPs) has been estimated to be 50% due to a lack of information about severe pathology, previous non-surgical treatment, and the duration of pain [14]. Efforts should focus on providing timely and warranted healthcare to those who need it, as emphasised by the Choosing Wisely initiative [12].

In this light, research-based interventions are needed to reduce the number of unwarranted MRIs. We implemented an intervention targeted at increasing the communication between the GP and the referral assessment team in the radiology department using correspondence letters to the GPs and an electronic referral guidance booklet.

In this study, we aimed to investigate the impact of the intervention on the appropriateness of referrals from GPs for lumbar MRI and the proportion of referred patients who underwent lumbar MRI.

### Setting

The Danish healthcare system is predominantly publicly funded through taxation. Danish residents have free access to hospitals and general practice, as well as partial reimbursement for some prescribed medications, physiotherapy, and chiropractic services [17].

In Denmark, nearly all residents are registered with a general practice, which serves as their first point of contact for medical care. General practitioners (GPs) function as gatekeepers to the broader healthcare system [17]. MRI and other diagnostic procedures conducted at public hospitals are available free of charge to Danish residents with a referral.

## Methods

### Study design

We employed a quasi-experimental design with defined pre- and post-intervention periods to evaluate the impact of the intervention.

The intervention commenced on 1 September 2022 and continued until 31 August 2023. The pre-intervention period from 1 January 2019 to 31 August 2022 was included for comparison. To guide the reporting of the intervention and the study methods, the template for intervention description and replication (TIDieR) [18] (Online Appendix 1) and the Strengthening the Reporting of Observational Studies in Epidemiology (STROBE) guideline [19] were applied.

### Study population

The study population consisted of all adult patients aged ≥ 18 years, listed with a general practice within the Central Denmark Region and with a referral to MRI of the lumbar spine at the Radiology Department, Silkeborg Regional Hospital, from their GP during 1 January 2019 to 31 August 2023.

### The intervention

MRI referrals from GPs were submitted to the hospital’s Radiology Information System (RIS), where they were assessed by the radiology referral assessment team. During the pre-intervention period, MRIs were generally initiated without further delay unless the referral lacked information on contraindications related to non-MRI-compatible materials.

The intervention consisted of standardised electronic correspondence letters that included information aligned with referral guidelines [8]. In addition, all GPs received an electronic referral guidance booklet outlining appropriate indications for lumbar spine MRI referrals in cases of LBP [13].

During the post-intervention period, referrals were evaluated as either appropriate or inappropriate based on the American College of Radiology (ACR) Appropriateness Criteria for LBP [8] (Online Appendix 2). The assessments were made with substantial to almost perfect inter-rater reliability [14, 20].

Referrals were categorised as appropriate if they included information indicating that the effect of relevant non-surgical treatment had been insufficient, or if they included information on suspicion of serious underlying pathology (fracture, malignancy, and infection), previous back surgery, candidate for lumbar spine surgery or suspected cauda equina. If a referral was deemed appropriate, the radiology department proceeded with the MRI.

Referrals were considered inappropriate if they lacked sufficient information to assess the indications listed above [13, 14]. Also, the referral assessment team noted whether inappropriate referrals lacked information on previous non-surgical treatment, the duration of non-surgical treatment, or other relevant details, e.g., missing narrative descriptions or referrals for other regions such as the cervical or thoracic spine.

If a referral was deemed inappropriate, a correspondence letter was sent to the GP explaining how the referral did not conform to the guidelines for referral. Template-based correspondence letters were integrated into the Electronic Patient Record (EPR) system and sent electronically by the referral assessment team, with content tailored to the specific shortcomings of the referral. In cases of uncertainty regarding the referral, real-time peer-to-peer telephone consultations between the radiology referral assessment team and the GP were conducted. The referral was returned if the GP had not responded within four days from the time the correspondence letter was sent.

### Data collection

Data were collected from the Data Warehouse of the Central Denmark Region. Study participants were identified through their personal identification number, assigned to all Danish residents, enabling linkage of information across registration systems in Denmark.

Information on age and gender was identified from the personal identification number. Information on marital status enters the Data Warehouse from the Civil Registration System. The Charlson Comorbidity Score [21] was calculated using diagnoses from the electronic patient record system, which contains information on patients admitted in the region. Radiological information about referrals and imaging enters the hospital’s Radiology Information Systems (RIS), from where lumbar spine MRI referrals and performed imaging were identified. Information about hospital contacts was obtained from the electronic patient records, and general practice contacts were collected from the LUNA System, which is used for administration and remuneration in the primary care sector. Data regarding whether a referral was deemed appropriate or not was assessed and registered by the radiology referral assessment team in a secure platform for research data entries; The Research Electronic Data Capture (REDCap) platform [22]. The pre-intervention assessments were made retrospectively, while post-intervention assessments were made prospectively and consecutively from the time of intervention start.

### Outcomes

*The proportion of appropriate referrals* was calculated monthly as the ratio of appropriate referrals for lumbar spine MRI out of the total number of lumbar spine MRI referrals. Post-intervention, the assessments were made real-time, while assessments for the pre-intervention periods were performed retrospectively based on medical records.

*The proportion of MRIs and radiographs* was calculated monthly as the ratio of lumbar spine MRIs and radiographs performed within 90 days of referrals for lumbar spine MRI out of the total number of referrals to lumbar spine MRI.

*The proportion of rejections* was found as the proportion of lumbar spine MRIs being rejected by the radiology referral assessment team or for which the GP had not responded within four days from the time the correspondence letter was sent.

*The proportion of re-referrals* was defined as the proportion of new referrals within 30 days of a rejected lumbar spine MRI referral. Re-referrals were categorised as appropriate or inappropriate, in line with the other referrals.

### Statistical analyses

Descriptive statistics were applied to describe the study population according to the pre- and post-periods and compared using Fisher’s exact test, Chi^2^, and Mann-Whitney U-test. Changes in the overall proportion of referrals for lumbar spine MRI, referral rejections, and re-referrals between periods were tested using the Mann-Whitney U-test.

Interrupted time-series analysis was employed using consecutive monthly data to evaluate the impact of the intervention. Some individuals had multiple referrals within short time intervals. Therefore, only the first referral per individual within one month was counted. The interrupted time-series analysis was conducted using Stata’s itsa command, which implements a segmented regression model with Newey–West standard errors to adjust for potential autocorrelation and heteroskedasticity.

The study period overlapped with the COVID-19 pandemic, which may have acted as a concurrent event potentially affecting outcomes. Also, the intervention was expected to have an adaptation period. To account for these potential biases, a sensitivity analysis was performed, censoring the period from March 2020 to December 2020 (COVID-19) and September 2022 to December 2022 (intervention adaptation period). In this, 1 February 2023 was set to evaluate slope change (time after intervention adaptation period) (Online Appendix 3).

All analyses were performed using a 5% significance level using Stata version 18.0.

## Results

In total, 5222 referrals to lumbar spine MRI from general practice were received by the radiology department at Silkeborg Regional Hospital during the study period. Of these, 4207 (80.6%) referrals were made in the pre-intervention period, and 1015 (19.4%) in the post-intervention period, corresponding to 95.6 and 84.6 per month, respectively. All studied patient characteristics were similarly distributed between the pre- and post-intervention periods (Table 1). Over half of those referred were females (pre: 58.0% and post: 59.5%), and referrals covered all age groups (18–29 years: 6.4%, 30–49 years: 29.8%, 50–64 years: 29.6%, 65 + years: 34.2%).

**Table 1:** Characteristics of adults referred to MRI of the lumbar spine in the Central Denmark Region during 1 January 2019 to 31 August 2022 (pre-intervention) and 1 September 2022 to 31 August 2023 (post-intervention)

	Total		Pre intervention			Post intervention			*p*-value	
	*N*	%	*N*		%	*N*		%		
Total referrals	5222	100	4207		100	1015		100		
Referrals of females	3043	58.3	2439		58.0	604		59.5	0.395	
Referrals by age groups 18–29 year 30–49 years 50–64 years 65 + years	335 1556 1543 1788	6.4 29.8 29.6 34.2	263 1261 1247 1436		6.3 30.0 29.6 34.1	72 295 296 352		7.1 29.1 29.2 34.7	0.737	
Referrals according to marital status Married/registered with a partner Widower/divorced/unmarried	2872 2350	55.0 45.0	2330 1877		55.4 44.6	542 473		53.4 46.6	0.254	
Utilisers of private practice physiotherapy 1 year before referral	551	10.6	417		9.9	134		13.2	0.219	
Charlson Comorbidity Score > 0	1293	24.8	1058		25.2	235		23.2	0.195	
Admissions 1 year before referral > 0	858	16.4	682		16.2	176		17.3	0.396	
Days with admission 1-year before referral 1 2–5 >5	431 261 166	8.3 5.0 3.2	339 209 134		8.1 5.0 3.2	92 52 32		9.1 5.2 3.2	0.760	

	Median	IQR	Median		IQR	Median		IQR	*p*-value	
Outpatient visits 1 year before referral	4	(2–8)	4		(2–8)	3		(2–7)	0.288	
GP contacts 1 year before referral	11	(5–20)	11		(5–20)	11		(5–20)	0.796	

GP contacts include daytime consultations, emails and telephone consultations. GP, general practitioner; IQR, interquartile range; MRI, magnetic resonance imaging

**Fig. 1:**
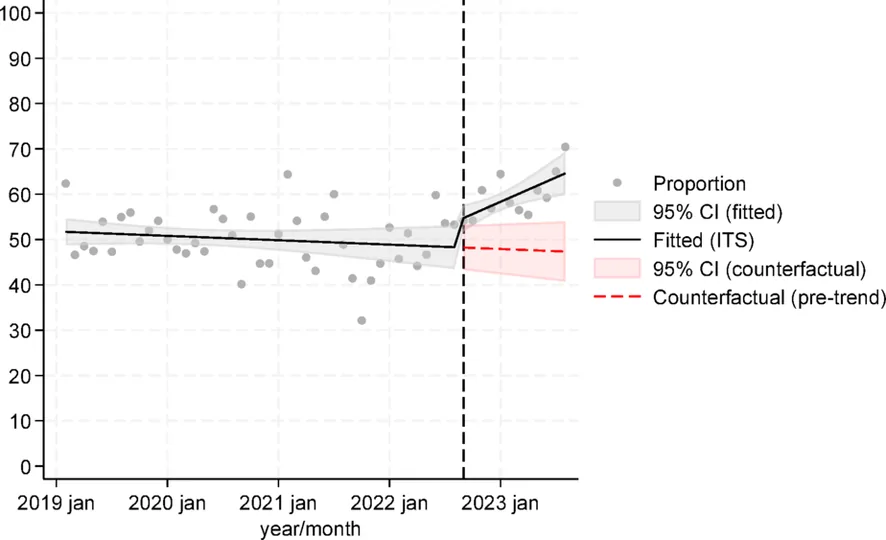
Proportion (%) of lumbar spine MRI referrals with appropriate indication out of all lumbar spine MRI referrals by GPs. The dashed line represents the onset of the intervention. CI, confidence interval; GPs, general practitioners; MRI, magnetic resonance imaging; ITS, interrupted time series

Overall, the proportion of appropriate referrals increased from 49.7% pre- to 60.0% post-intervention (*p* < 0.001) (Table 2). The interrupted time series regression analysis did not demonstrate a statistically significant increase in the proportion of appropriate referrals at the start of the intervention (level change = 6.50 (95% CI 0.71, 12.29), *p* = 0.028). However, the slope changed from the pre- to post-intervention period (slope change = 0.98 (95% CI 0.36, 1.59), *p* = 0.002) (Table 3 and Fig. 1). This indicated that the improvement in appropriate referrals continued over time.

**Table 2:** Referrals of adults to MRI of the lumbar spine in the Central Denmark Region during 1 January 2019 to 31 August 2022 (period without intervention) and 1 September 2022 to 31 August 2023 (period with intervention)

		Total			Pre interventio		Post intervention				*p*-value
		*N*	%		*N*	%	*N*		%		
Lumbar spine MRI referrals		5222	100		4207	100	1015		100		
Referrals with indication (deemed appropriate) Referrals without indication		2701 2521	51.7 48.3		2092 2115	49.7 50.3	609 406		60.0 40.0		< 0.001
Reasons for ‘referrals without indication’: No information about non-surgical treatment Insufficient information about the time aspect^A^ Other reasons^B^ or missing		989 1226 306	39.2 48.6 12.1		822 1009 284	38.9 47.7 13.4	167 216 23		41.1 53.2 5.7		

MRI, magnetic resonance imaging. Referrals with indication: the number of lumbar spine MRI referrals deemed appropriate, as they were assessed to conform with national guidelines. Additional referrals for the same individual within 30 days were not included. ^A^ Referral earlier than 6 weeks from symptom start. ^B^ N = 5, in the pre-intervention period

**Table 3:** Coefficients, level change, and slope change for the proportion of appropriate lumbar spine MRI referrals and for the proportion of lumbar MRIs performed out of all referrals, pre and post intervention, corresponding to Figs. 1 and 2

*MRI referrals with indication*			
	Coefficient	95% CI	*p*-value
Pre-intervention slope	− 0.08	(− 0.24, 0.08)	0.328
Post-intervention slope	0.90	(0.30, 1.49)	0.003
	Change in %	95% CI	*p*-value
Level change at onset of the intervention	6.50	(0.71, 12.29)	0.028
Slope change pre versus post intervention	0.98	(0.36, 1.59)	0.002
*MRI scans performed*			
	Coefficient	95% CI	*p*-value
Pre-intervention slope	− 0.12	(− 0.26, 0.02)	0.100
Post-intervention slope	0.66	(− 0.05, 1.37)	0.067
	Change in %	95% CI	*p*-value
Level change at onset of the intervention	− 17.06	(− 23.25, − 10.88)	< 0.001
Slope change pre versus post intervention	0.79	(0.05, 1.51)	0.036

CI, confidence interval; MRI, magnetic resonance imaging

**Fig. 2:**
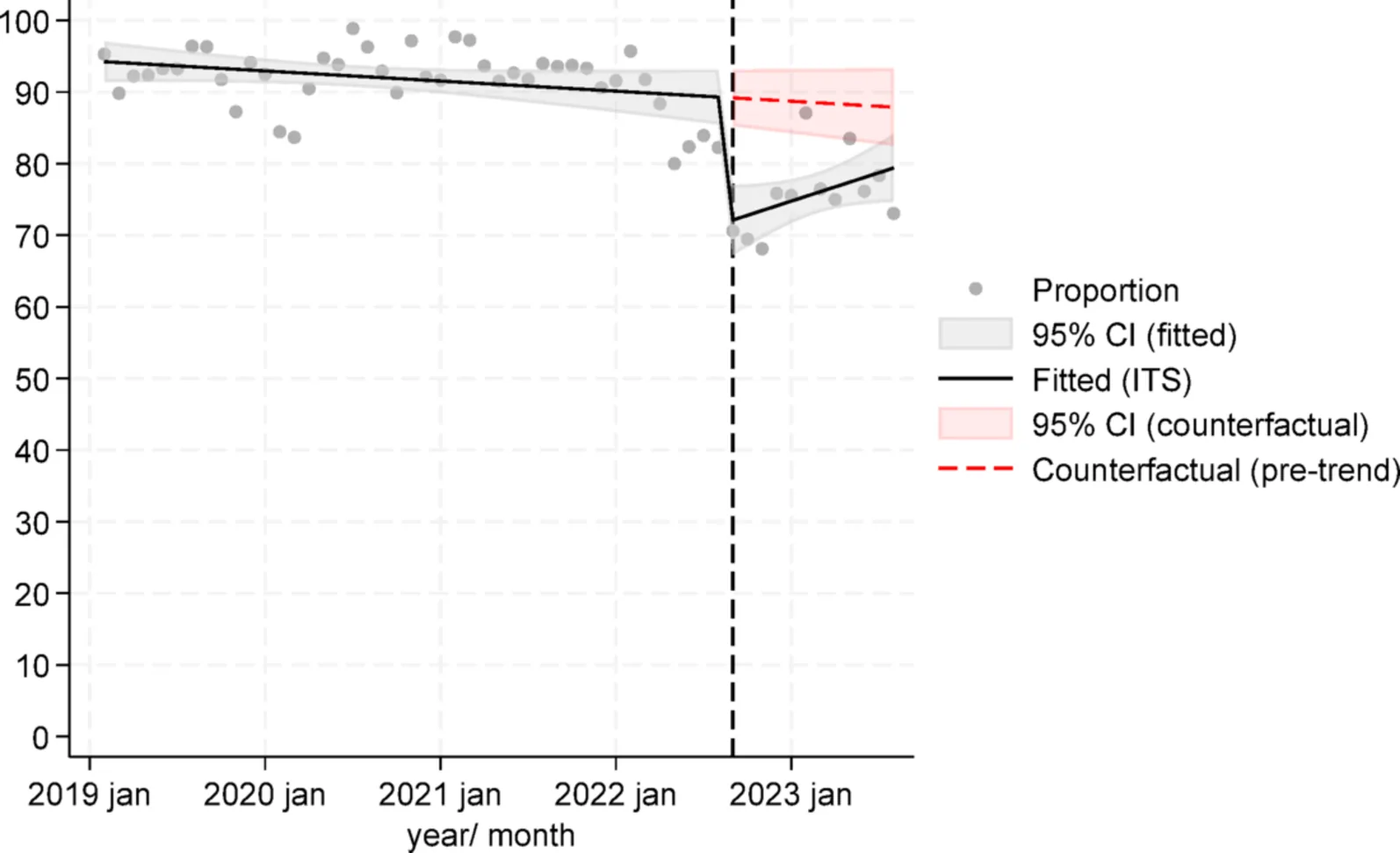
The proportion (%) of lumbar spine MRI performed out of all GP referrals to lumbar spine MRI. The vertical dashed line represents the onset of the intervention. CI, confidence interval; GP, general practitioner; MRI, magnetic resonance imaging; ITS, interrupted time series

The proportion of lumbar spine MRIs performed within 90 days of a referral decreased pre- versus post-intervention from 92.2% (*n* = 3879) to 75.7% (*n* = 768) (*p* < 0.001). A significant immediate slope drop was observed at the onset of the post-intervention period, with a level change of − 17.06% points (95% CI − 23.25, − 10.88)(*p* < 0.001). However, we observed a statistically significant positive slope change of 0.79 (95% CI 0.05, 1.51) (*p* = 0.036) implying that the post-intervention proportion could be projected to converge towards the pre-intervention level over time (Table 3 and Fig. 2). Therefore, over time, the proportion could return to the level it was before the intervention.

In the post-intervention period, 214 unique referrals for lumbar spine MRI were rejected, corresponding to 21.1% of all lumbar spine referrals and 52.7% of those classified as inappropriate. Re-referrals submitted within 30 days after a rejection (*n* = 60) accounted for 5.6% of rejected referrals in the post-intervention period.

Post hoc analysis of lumbar spine radiographs performed within 90 days of a lumbar MRI referral showed a decrease from 2.5% (*n* = 104) in the pre-intervention period to 0.3% (*n* = 3) post intervention (*p* < 0.001).

In the sensitivity analysis, censoring for COVID-19 and wash-in, a statistically significant slope change and level change were seen for appropriateness of lumbar spine MRI referrals. A statistically significant change was found for the level change (after wash-in) and not for the slope change for the proportion of lumbar spine MRIs performed (Online Appendix 3).

## Discussion

### Main results

After introducing standardised electronic correspondence letters and an electronic guidance booklet, we found a significant increase in the proportion of lumbar spine referrals for MRI from general practice that were deemed appropriate by the radiology referral assessment team, indicating potential impact in enhancing guideline adherence. However, 40% of referrals remained non-compliant with guidelines, despite the intervention. The intervention reduced the number of lumbar MRIs performed, although possibly without sustained effect.

### Comparisons with the literature

Studies have showed that clinical guidelines for managing LBP are perceived by clinicians as categorical, overly prescriptive, and limiting to clinical judgement and professional autonomy [23–25], which may prevent their application in clinical practice.

Several studies have explored the appropriateness of lumbar spine MRI referrals [14, 26–29]. In a systematic review, Jenkins et al. found that inappropriate imaging is frequent in LBP management, including both overuse in patients where imaging is not indicated as well as underuse of imaging, when it is indicated [28]. Research from our own group has shown that up to 50% lumbar spine MRI referrals from GPs were deemed inappropriate based on the information available in the referral and weighed against the ACR Imaging Appropriateness Criteria [14]. A prospective multicentre study in Spain found that 43% of lumbar spine MRI referrals were classified as inappropriate [26]. Also, an Iranian study by Jahanmehr et al. reported that 56.7%, 39.7%, and 3.6% of lumbar MRI referrals made at public hospitals were appropriate, inappropriate or uncertain, respectively [30]. In the UK, Hudson et al. showed that 46% of referrals were considered clinically justified, 36% were unjustified and 17% were questionable [31]. Thus, these studies support a need to optimise referral practice for lumbar spine MRI in patients with LBP. Some control regarding the referral practice may be necessary to reduce the use of MRI services. For example, a natural experiment in the Southern Denmark Region demonstrated that direct access to lumbar MRI for GPs and chiropractors increased MRI rates by 115% from 2010 to 2013, compared with other Danish regions [27].

Efforts to reduce inappropriate lumbar spine MRI referrals have been found effective, including clinical decision support tools, structured referral forms, and checklists [32–35]. These initiatives demonstrate a proactive approach to improving MRI referral appropriateness. In Australia, a 12-weeks multifaceted implementation strategy with a combination of education, feedback, and decision support was designed to improve concordance between clinical practice and guidelines for the management of low back pain. The intervention resulted in only modest changes in general practice referral behaviour [14]. Ip et al. demonstrated the effectiveness of a multifaceted clinical decision-support intervention in adult primary care patients with LBP. The intervention included a structured peer-to peer consultation and computerised practice pattern variation report, which significantly reduced the utilisation of lumbar spine MRI [32]. Other interventions to reduce lumbar spine referrals include clinical education, which has shown to reduce the number of MRIs performed for uncomplicated LBP and to improve MRI appropriateness [36]. Alanazi et al. applied machine learning algorithms to develop vetting models checking referrals’ eligibility for lumbar spine MRI, ensuring compliance with international guidelines [37]. Belavy et al. conducted a systematic review of interventions to improve guideline-recommend imaging referrals in LBP. Including eight studies, Belavy et al. found low-certainty evidence that interventions to reduce imaging referrals had no effect. The authors argued that clinician education and passive dissemination (i.e., guidelines) were, in isolation, likely inefficient approaches [38]. Our intervention employed the ACR Imaging Appropriateness Criteria to assess appropriateness, provided structured feedback through standardised electronic correspondence letters. It also incorporated a clinical decision support tool in the form of an electronic guidance booklet. This makes the intervention broad and similar to earlier interventions that used referral guidelines/checklists, clinical decision support tools, or structured feedback. In a review conducted by Kamper et al. on back pain care, findings revealed that around 25% underwent unnecessary imaging referrals, highlighting a continued need for additional solutions.

In conclusion, although clinical guidelines for lumbar spine MRI referrals are well established, inappropriate referrals remain common. Studies have highlighted the need for adherence to guidelines and prompted efforts to reduce unwarranted imaging. Still, only few interventions systematically assess referral appropriateness, highlighting an ongoing challenge in ensuring that patients receive appropriate and effective care.

### Strengths and limitations

This quasi-experimental study included all individuals referred to lumbar MRI at a Danish hospital and had statistical precision due to the large the study population. Due to the electronic registration, which routinely takes place during clinical practice, our data can be considered highly valid and complete.

Some limitations merit consideration. The pre-post study design is at risk of producing conclusions based on temporal trends, which may affect the validity, sustainability and generalisability of the findings. The intervention period ended after a duration of only 12 months due to organisational changes within the hospital. It seems unlikely that seasonal variation between the pre- and post-intervention periods had a high impact on the observed results. Although the intervention showed effectiveness, a larger proportion of referrals remained inappropriate, and the intervention was not followed for a sufficient after-period to determine a sustained impact, which the sensitivity analysis supported. The inclusion of a wash-in period analysis and exclusion of COVID-19 period in the sensitivity analysis helped reduce potential bias but with the compromise of few data entries.

Assumptions that similar intervention benefits will apply to other hospital settings should be made with caution. Generalisation requires health care systems that resemble the Danish, where referral for imaging is GP-initiated and subjected to guidelines.

### Clinical and research perspectives

Within the previous decade, the utilisation of lumbar spine MRIs has increased by 25% in Denmark [39]. Several studies have demonstrated the downstream consequences of inappropriate lumbar spine MRI referrals, including increased rates of lumbar spine interventions and elevated healthcare costs [35, 40], placing a substantial burden on healthcare systems [2, 41, 42]. When public healthcare services are perceived as insufficient or inaccessible, patients may turn to private healthcare providers, which can exacerbate inequities in access to care. Alternatively, GPs may refer patients for MRI without sufficient clinical indication [16]. By improving the appropriateness of referrals, such unintended consequences may be mitigated. The Danish Resilience Commission mentions in its report on inappropriate treatment in the healthcare system that the GP’s gatekeeper function is challenged by high demand for referrals from, among others, private health insurance, as well as perceived pressure from patients and relatives [43].

The standardised correspondence letters and referral guidance booklet may have translated complicated guidelines into understandable referral practice, thus assisting GPs in more appropriate referrals.

Moreover, real-time peer-to-peer telephone consultations provided an additional layer of communication, ensuring that unclear cases received timely resolution. However, adverse effects are also possible. The initial reduction in MRI performed may reflect the intervention’s workflow, whereby returned referrals and the atypical nature of reissuing them may have temporarily limited GP engagement. This mechanism could account for the early decline, with the subsequent gradual increase reflecting growing familiarity and integration of the process into routine practice. Thus, the requirement for GPs to respond might have led to unintended delays in patient care. The manual nature of sending correspondence letters in our intervention suggests a potential administrative burden, which could be mitigated through automation.

Implementation of the intervention requires resources beyond those needed for reviewing referrals alone. Beyond a start-up phase, it introduces additional complexity by requiring an assessment of relevance and a corresponding response and dialogue with general practice, thereby increasing time demands. This may translate into greater staff-hours, depending on workflow organisation and case volume. Implementation will also depend on adequate system support; adapted to existing digital or administrative platforms. Finally, engagement and endorsement from hospital leadership are essential to support prioritisation, resource allocation, and sustained integration into routine practice.

Further studies are needed to evaluate the sustainability of the intervention across diverse settings, ideally employing a randomised controlled trial design. Future studies should also focus on the implementation processes and contextual factors that may influence the intervention’s effectiveness and scalability.

## Conclusion

Aligned with the principles of the Choosing Wisely initiative for LBP, this study suggests that the use of standardised correspondence letters combined with an electronic referral guidance booklet may support clinicians by providing relevant information and clinical indicators in the referrals, thereby potentially reducing inappropriate referrals and lumbar spine MRIs.

## Supplementary Information

Below is the link to the electronic supplementary material.

Supplementary Material 1.

Supplementary Material 2.

Supplementary Material 3.

## Data Availability

The dataset used in the current study is available from the corresponding author on reasonable request.

## Abbreviations

CI: Confidence interval
CT: Computed tomography
GP: General practitioner
LBP: Low back pain
MRI: Magnetic resonance imaging
