# Supplementary material for: Reducing inappropriate lumbar MRI referrals: a Danish pre- and post-intervention study
Source: Chiropr Man Therap. 2026 Jul 4;34:52. doi: 10.1186/s12998-026-00663-x (PMC13548564; doi:10.1186/s12998-026-00663-x)
Supplement: Supplementary file 1 [file 12998_2026_663_MOESM1_ESM.docx]

# Appendix 1. TIDieR Checklist – Intervention Description

| **Item** | **Description** |
| --- | --- |
| 1. Brief name | Standardised correspondence and referral guidance intervention for lumbar spine MRI referrals. |
| 2. Why | The intervention aimed to improve the appropriateness of lumbar spine MRI referrals by ensuring alignment with Danish national referral guidelines and enhancing communication between radiologists and general practitioners (GPs). |
| 3. What – materials | - Standardised electronic correspondence letters integrated into the Electronic Patient Record (EPR). - An electronic referral guidance booklet sent to all GPs, outlining indications for lumbar spine MRI in low back pain (LBP) cases. |
| 4. What – procedures | - Referrals submitted via the Radiology Information System (RIS) were assessed by the radiology visitation team. - If the referral was appropriate, MRI was scheduled. - If inappropriate or missing essential information, a standardised correspondence letter was sent to the GP. - Letters were tailored based on missing details (e.g., prior non-surgical treatment, unclear duration, vague indications). - Peer-to-peer phone consultations occurred in cases of uncertainty. - Referrals were rejected if the GP did not respond within four days of the correspondence letter being sent. |
| 5. Who provided | The intervention was delivered by the hospital’s radiology visitation team. |
| 6. How | - Correspondence letters were sent electronically via the EPR system. - Real-time peer-to-peer consultations via telephone. - Referral guidance booklet was distributed electronically to all GPs. |
| 7. Where | The intervention took place at a single hospital radiology department, in Central Denmark Region, Denmark. GPs interacted remotely from their practices. |
| 8. When and how much | The intervention commenced 1 September 2022 for all lumbar spine MRI referrals. |
| 9. Tailoring | Yes – the content of correspondence letters was template-based but customised depending on what was missing or unclear in the referral. |
| 10. Modifications | No. |
| 11. How well – planned fidelity assessment | Referrals were assessed prospectively during the post-intervention period and assessed in real-time.  Referrals in the pre-intervention period were assessed historically. Both periods had 100% completeness for assessment. A former study by Krogh et al. showed almost perfect inter-rater reliability (13, 18). No other fidelity assessments were planned. |
| 12. Actual fidelity | 100% completeness on assessment for appropriateness, whether a correspondence letter were sent in case of an inappropriate referral, was not assessed. |
