# Supplementary material for: Reducing inappropriate lumbar MRI referrals: a Danish pre- and post-intervention study
Source: Chiropr Man Therap. 2026 Jul 4;34:52. doi: 10.1186/s12998-026-00663-x (PMC13548564; doi:10.1186/s12998-026-00663-x)
Supplement: Supplementary file 2 [file 12998_2026_663_MOESM2_ESM.docx]

**Appendix 2**.

Flowchart for the classification of referrals according to the ACR Imaging Appropriateness Criteria

*Adapted from*

*Krogh, S.B., Jensen, T.S., Rolving, N. et al. Appropriateness of referrals from primary care for lumbar MRI. Chiropr Man Therap 30, 9 (2022). https://doi.org/10.1186/s12998-022-00418-4_*
