# Supplementary material for: Reducing inappropriate lumbar MRI referrals: a Danish pre- and post-intervention study
Source: Chiropr Man Therap. 2026 Jul 4;34:52. doi: 10.1186/s12998-026-00663-x (PMC13548564; doi:10.1186/s12998-026-00663-x)
Supplement: Supplementary file 3 [file 12998_2026_663_MOESM3_ESM.docx]

**Appendix 3**

| *Changes excluding the covid period (Marts 2020 to December 2020) and the wash-in period (September 2022 to February 2023): Coefficients, level change, and slope change for the proportion of appropriate lumbar spine MRI referrals and for the proportion of lumbar MRIs performed out of all referrals, pre and post intervention.* | | | |
| --- | --- | --- | --- |
| **MRI referrals with indication** |  |  |  |
|  | **Coefficient** | **95% CI** | **p-value** |
| Pre-intervention slope | -0.13 | (-0.37, 0.12) | 0.307 |
| Post-intervention slope | 2.90 | (1.97, 3.83) | <0.001 |
|  | **Change in %** | **95% CI** | **p-value** |
| Level change (after wash-in) | 6.16 | (0.29, 12.03) | 0.040 |
| Slope change pre vs. post intervention | 3.02 | (1.98, 4.06) | <0.001 |
| **MRI scans performed** |  |  |  |
|  | **Coefficient** | **95% CI** | **p-value** |
| Pre-intervention slope | -0.22 | (-0.42, -0.027) | 0.025 |
| Post-intervention slope | -0.19 | (-1.35, 0.96) | 0.744 |
|  | **Change in %** | **95% CI** | **p-value** |
| Level change (after wash-in) | -9.53 | (-15.15, -3.90) | 0.001 |
| Slope change pre vs. post intervention | 0.03 | (-1.19, 1.25) | 0.963 |

*Abbreviations: CI: Confidence Interval, MRI: Magnetic Resonance Imaging*
